# Functional feed ingredients modulate the immune response of RTgutGC cells to LPS-induced inflammation

**DOI:** 10.3389/fimmu.2025.1616076

**Published:** 2025-06-18

**Authors:** Malene Malchus Fosse, Laura Rivera Méndez, Tania Rodríguez-Ramos, Brian Dixon, Henrik Sundh, Rolf Erik Olsen

**Affiliations:** ^1^ Department of Biology, Norwegian University of Science and Technology, Trondheim, Norway; ^2^ Department of Biology, University of Waterloo, Waterloo, ON, Canada; ^3^ Department of Biological and Environmental Sciences, University of Gothenburg, Gothenburg, Sweden

**Keywords:** β-glucans, bioactive peptides, RTgutGC, fish gut health, lipopolysaccharide (LPS), immune regulation

## Abstract

Functional feed ingredients can enhance the fish gut integrity and immune resilience during challenging situations in the aquaculture industry. This study used the RTgutGC cell line – derived from rainbow trout intestinal epithelium, to evaluate the immunomodulatory and barrier effects of selected ingredients. These included β-glucan from *Saccharomyces cerevisiae* (BG40 and BG60), laminarin extracted from *Laminaria hyperborea* (Lam60 and Lam90), and bioactive peptides with antioxidative and immunomodulatory potential; carnosine (Carn100 and Carn120) and salmon hydrolysate (FPH300 and FPH600). Cells were exposed for 24 hours at two concentrations (maintaining 100 % and 80 % viability), and effects on transepithelial resistance (TEER), permeability (P_app_) and gene expression (qPCR) were assessed before and after a 6-hour lipopolysaccharide (LPS) challenge. High-dose laminarin and both salmon hydrolysate concentrations elevated mRNA encoding for pro-inflammatory cytokines (*il6*, *il8*, *il1b* and *tnfa*; *p* < 0.05). All ingredients except carnosine significantly reduced TEER (*p* < 0.05) often with downregulation of barrier genes. Low-dose carnosine and laminarin reduced P_app_ of Lucifer yellow, indicating less barrier disruption. LPS induced inflammation, barrier dysfunction and reduced proliferation. These effects were modulated by high-dose β-glucan and both laminarin concentrations, which significantly reduced *il6* expression (*p* < 0.05). High-dose salmon hydrolysate also tended to reduce *il6* (*p* = 0.05) and increased *pcna* expression (*p* < 0.001), suggesting tissue recovery. Low-dose laminarin and both carnosine concentrations upregulated *cldn3* post-challenge (*p* < 0.05). These findings support the RTgutGC model as a valuable screening tool and provides new insights into the biological activity and immunomodulatory effects of various functional feed ingredients.

## 1 Introduction

The fish gut is essential for digestion, nutrient absorption, and osmoregulation, while also playing a critical role in maintaining immune homeostasis (1–3). A crucial component of this defense is the intestinal epithelium, which serves as both a physical and immunological barrier (4). Tight junction proteins, such as zonula occludens (ZO-1), occludins, and claudins, regulate paracellular permeability controlling the selective passage of ions, nutrients, and water (1). In aquaculture, however, gut health is increasingly challenged by reduced availability of marine-based ingredients and challenging farming practices (5–11). These ingredients and stressors can compromise the integrity of the intestinal barrier, increasing permeability and leaving the fish more vulnerable to infections and diseases (2, 6, 11–17). Originally, antibiotics were used in Norway to control diseases in fish farming and are still used in some countries. However, this approach raised concerns about antibiotic resistance and its negative impact on fish health and the environment (18). With the development of vaccines, a significant proportion of common pathogenic diseases has been effectively controlled. However, substantial challenges remain concerning the complex interactions between environmental stressors, physiological stress and antinutritional factors, which can compromise the immune function and increase susceptibility to opportunistic pathogens and other infections in fish (19). As a result, functional feed ingredients, including prebiotics, probiotics, β-glucans and peptides, have received increasing attention for their potential to boost fish health, enhance immunity and offer a more sustainable alternative to antibiotics (9, 20–25).

β-glucans are polysaccharides composed of β-(1,3)-linked β-_D_-glucopyranosyl backbone with β-(1,6)-linked side chains of varying distribution and lengths. They are key structural components of the cell wall of bacteria, algae, fungi and plants where they play essential roles in defense mechanisms and energy storage (26). β-glucans are widely recognized as potent immune stimulators, with demonstrated benefits on promoting growth, gut health, immune responses and disease resistance in fish (27, 28). The high molecular weight β-glucan derived from the cell wall of baker’s yeast (*Saccharomyces cerevisiae*) is the most extensively studied in aquaculture, but in recent years, lower molecular weight β-glucans extracted from brown seaweeds of the *Laminaria* genus (commonly referred to as laminarin, LAM) have gained attention (29–31).

In addition to β-glucans, bioactive peptides have emerged as another class of functional feed ingredients with promising benefits for fish health. These peptides, derived from protein hydrolysis, possess diverse biological activities, including antimicrobial, antioxidant and immunomodulatory effects (32, 33). One such peptide, carnosine, is a naturally occurring dipeptide composed of β-alanine and L-histidine (34). Although studies of the effect of carnosine in fish is limited, it has shown to have antioxidant and immunomodulatory effects in rodents and in human intestinal Caco-2 cells, making it a potential candidate for improving disease resistance (35, 36). A significant source of bioactive peptides in aquafeeds is found in fish protein hydrolysate (FPH), a protein-rich byproduct obtained from enzymatic hydrolysis of processing waste (37). FPH contains a complex mixture of peptides with demonstrated effects on gut health, nutrient absorption and immune modulation in farmed fish species (38–42).

*In vitro* models, such as intestinal epithelial cell lines, are invaluable for targeted research on the effects of functional feed ingredients on the epithelial immune response and barrier function of the fish gut. These models not only align with the 3R principle by reducing the need for experimental animals, but also offer a more cost-effective, faster, and less labor-intensive screening tool for the compounds of interest. Until recently, the RTgutGC cell line, derived from the distal intestine of a small female rainbow trout (*Oncorhynchus mykiss*), was the only immortalized cell line available from fish gut tissue (43). When cultured as a monolayer in a two-compartment barrier model—with the apical compartment simulating the intestinal lumen and the basolateral compartment simulating the portal blood side—RTgutGC cells form a polarized epithelium characterized by tight junctions, cell-to-cell adhesions, and measurable transepithelial resistance (44, 45). This cell line has been extensively characterized since its establishment and has been a valuable tool for studying nutrient uptake, toxicity, functional feed ingredients, gut immune function, and epithelial integrity in fish (43, 45–50). RTgutGC cells have also been shown to respond to the bacterial endotoxin lipopolysaccharide (LPS) exposure with elevated gene expression levels of pro-inflammatory cytokines (43, 48, 49). The introduction of immune challenges, such as exposure to LPS, can mimic pathogen-induced stress, providing valuable insights into the immune responses of the gut’s epithelial cells and the interplay between barrier integrity and inflammation.

Maintaining a functional intestinal barrier and immune response is critical for disease resistance, growth performance and overall well-being in farmed fish (9, 11, 12, 19, 24). In the search for functional feed ingredients to support fish intestinal health, the present work aimed to investigate: a) how treating RTgutGC cells with two different concentrations of glucans; β-glucan from baker’s yeast (*S. cerevisiae*) and laminarin extracted from *L. hyperborea*, and two different concentrations of bioactive peptides; carnosine and salmon hydrolysate, for 24 hours impacted immune- and barrier function under normal conditions and b) how pretreatment with these functional feed ingredients would impact the RTgutGC cells in response to a 6-hour exposure of LPS.

## 2 Materials and methods

### 2.1 Routine cultivation of the RTgutGC cell culture

The RTgutGC cell line was kindly gifted by Tania Rodríguez-Ramos and Prof. Brian Dixon (University of Waterloo, Canada). Routine cultivation followed the protocol described by Kawano et al., 2011. In brief, the cells were grown in 75 cm^2^ culture flasks (13-680-59, Fisher Scientific) containing Leibovitz’s L-15 complete medium (L-15/C) with Phenol red and 2.05 mM L-glutamine (L1518, Sigma-Aldrich) supplemented with 10 % fetal bovine serum (FBS) (97068-085P, Avator) and 1 % Penicillin-Streptomycin (P/S) (P4333, Sigma-Aldrich). The cells were cultured at 20°C in an incubator with a normal atmosphere, and the media was changed twice weekly. Upon reaching confluency, determined by visually inspecting the flask using an inverted microscope, the cells were pretreated with 0.8 mL Versene solution (15040066, Gibco, Thermo Fisher) for 5 min to facilitate dissociation, then trypsinized with 0.25 % trypsin in PBS without Ca^2+^ and Mg^2+^ (392-0436, VWR) for 2 min. Confluency was typically reached after 7–9 days and the cells were subsequently sub-cultured in a 1:2 ratio.

### 2.2 Preparation of the functional feed ingredients

#### 2.2.1 Stock solution preparation

Stock solutions for the functional ingredients and bacterial LPS challenge were prepared shortly before use and stored at -20°C. The LPS stock solution (L2630, VWR) was prepared at 1000 µg/mL in L-15/ex. β-glucan from baker’s yeast *S. cerevisiae* (G5011, Sigma-Aldrich) was prepared to 1000 µg/mL in sterile PBS, following established protocols (51). The β-glucan solution was sonicated in a water bath at 60°C in 30-second pulses until fully dissolved. Laminarin with purity of >95 % was extracted from *Laminaria hyperborea* by Ingeborg Hollekim Bringslid using acid assisted hot water extraction and was prepared to 1000 µg/mL in sterile PBS. Salmon hydrolysate (FPH) was processed from fresh salmon offal by Nutrimar AS and was prepared at 2000 µg/mL in L-15/ex. The composition analysis, performed by Eurofins according to Sandbakken et al., 2023 is listed in **Supplementary Table 1**. L-Carnosine (C9625, Sigma-Aldrich) was prepared at a concentration of 500 mmol/L in distilled water to ensure complete solubility (52). Working solutions were freshly prepared from the stock solutions immediately before use by diluting them in L-15/ex.

#### 2.2.2 Cell viability assay for determination of working concentrations

A cell viability assay was conducted in 96-well plates without inserts (Corning, flat bottom) to determine the optimal working concentrations of the ingredients (**Supplementary Table 2**). This assay was conducted in two independent experiments with overlapping concentrations similar to the study of Wang et al., 2019. It was performed without the use of membrane inserts, assuming similar response with and without the membrane inserts. Briefly, cells were seeded at a density of 62500 cells/cm^2^ in 96-well plates, with 200 µL of cell suspension (1 x 10^5^ cells/mL) in L-15/C added to each well. After 48 hours of attachment and acclimation, the media was aspirated, and the cells were washed with 200 µL PBS per well. Subsequently, working solutions of the functional ingredients and LPS at various concentrations (**Supplementary Table 2**) were added in quadruplicate to their respective wells. The cells were then incubated with either the functional ingredients for 24 hours or LPS for 6 hours in 20°C. Alamar Blue (AB, DAL1025, Invitrogen) and 5-carboxyfluorescein diacetate acetoxymethyl ester (CFDA-AM, C1345, Invitrogen) were combined in L-15/ex to give final concentrations of 5 % (v/v) and 4 µM, respectively, following the method described by Schirmer et al., 1997. The AB assay assesses cell metabolic activity by measuring the redox potential of cultures, as only live cells can metabolically reduce the non-fluorescent resazurin into its highly fluorescent form and CFDA-AM is a cell-permeant substrate used to assess both cell membrane integrity and enzymatic activity (53). CFDA-AM requires enzymatic conversion to become fluorescent, and only cells with intact membranes can retain the activated substrate intracellularly, making it an effective indicator of membrane functionality.

After incubation with the working solutions, the media was aspirated, and the cells washed with 200 µL PBS. Then a 100 µl aliquot of the fluorescent dye mix was added to each well, followed by incubation in the dark at 20°C for 60 min. Fluorescence was measured using a microplate reader (VLB000 Varioskan™ LUX mulitimode) with excitation/emission wavelengths of 530/590 nm for AB and 494/541 nm for 5-CDFA-AM. Fluoresence readings were recorded as relative fluorescent units (RFUs) based on a single read. Background fluorescence from wells without cells (blank) was subtracted from both control and stimulated cells. RFU data were expressed as percentage viability compared to the control. Based on the result from the cell viability assay, a level maintaining 100 % and 80 % viability was chosen for each of the functional feed ingredients; β-glucan with 40 µg/mL and 60 µg/mL (BG40 and BG60), laminarin with 60 µg/mL and 90 µg/mL (Lam60 and Lam90), L-Carnosine with 100 mM and 120 mM (Carn100 and Carn120) and salmon hydrolysate with 300 µg/mL and 600 µg/mL (FPH300 and FPH600). For LPS, a concentration of 60 µg/mL was chosen.

### 2.3 Transwell culturing system

**Figure 1:**
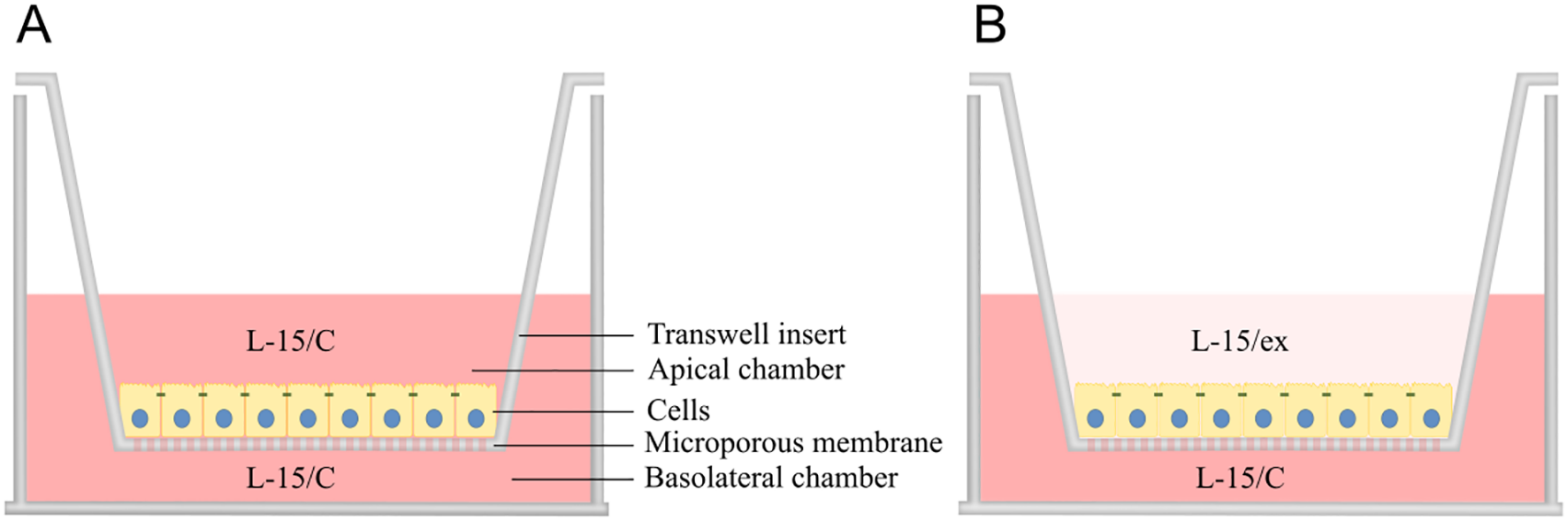
Two-compartment intestinal barrier model with transwell insert mimicking intestinal lumen (apical chamber) and portal blood (basolateral chamber) environment. After seeding, the cells were grown for ~3 weeks on the microporous membrane with Leibovitz L-15 complete (L-15/C) medium in both compartments to facilitate growth and forming of monolayer **(A)**, whereas L-15/C was replaced with reduced medium (L-15/ex) in the apical chamber during the experiment to more closely reflect the milieu of the intestinal lumen **(B)**. Illustration by: Malene Malchus Fosse.

The RTgutGC cells have demonstrated the ability to form a polarized epithelium when cultured on permeable supports in a two-compartment intestinal barrier model, as documented in previous studies (44, 45, 48). To mimic the intestinal lumen (apical/upper chamber) and portal blood (basolateral/lower chamber) environments, 24-well plates (734-0067, Falcon, Corning) with 33.6 mm^2^ transparent transwell inserts (734-0036, Falcon, Corning) and 6-well plates (734-0065, Falcon, Corning) with 425.4 mm^2^ transparent transwell inserts (734-0032, Falcon, Corning) with pore size 0.4 µm were utilized. Cells were counted using a Bright-Line Hemacytometer (Z359629 Merk) and seeded at a density of 62500 cells/cm^2^. Specifically, 300 µl of cell suspension at 68750 cells/mL was added to the apical chamber of 24-well inserts, and 3.5 mL of suspension at 80714 cell/mL was used for 6-well inserts. Before reaching confluency, the media in both compartments were filled with L-15/C (**Figure 1A**). To better resemble the low nutrient concentration in the intestinal lumen versus the high nutrient concentration in the blood stream, the L-15/C in the apical chamber was replaced by L-15/ex during the experiment (**Figure 1B**) (44). L-15/ex is a reduced medium containing only inorganic salts, galactose and pyruvate concentrations of L-15, as formulated by Schirmer et al., 1997. The basolateral chamber contained 1 mL and 3.5 mL media for 24- and 6-well plates, respectively. The media in both compartments were changed 2 times per week over a period of 21 days.

### 2.4 Measurement of transepithelial electrical resistance

Following seeding of RTgutGC cells in 24-well culture plates with membrane inserts, cells were allowed to attach and acclimate for 3 days prior to measurement of transepithelial electrical resistance (TEER). TEER measurements were performed on days 3, 7, 14 and 20 using an EVOM 3 Voltohmmeter and Endohm-6 electrode (World Precision Instruments, Berlin, Germany). This was conducted to confirm monolayer formation (data not shown) (45). On day 20, after TEER measurement, apical media was removed and replaced with L-15/ex containing functional ingredients in quadruplicate inserts. Following 24 hours of exposure to the functional ingredients 40 µg/mL and 60 µg/mL β-glucan (BG40 and BG60), 60 µg/mL and 90 µg/mL laminarin (Lam60 and Lam90), 100 mM and 120 mM L-Carnosine (Carn100 and Carn120) and 300 µg/mL and 600 µg/mL salmon hydrolysate (FPH300 and FPH600), TEER was measured again. Subsequently, cells were exposed to 60 µg/mL LPS for 6 hours and TEER was measured once more. Experimental setup is illustrated in **Supplementary Figure 1**. TEER values were calculated by subtracting the background resistance (membrane inserts without cells) from the inserts containing cells, and results were reported as Ω x cm^2^.

### 2.5 Lucifer yellow translocation assay

RTgutGC cells seeded in 6-well culture plates with membrane inserts were cultured for 20 days. Media was weekly changed in the apical and basolateral compartments. After this period, the functional feed ingredients; 40 µg/mL and 60 µg/mL β-glucan (BG40 and BG60), 60 µg/mL and 90 µg/mL laminarin (Lam60 and Lam90), 100 mM and 120 mM L-Carnosine (Carn100 and Carn120) and 300 µg/mL and 600 µg/mL salmon hydrolysate (FPH300 and FPH600) were added to the six replicate wells. After a 24-hour incubation, the media was removed from three of the replicates per treatment. The apical membrane was washed with 1 mL PBS before 3 mL Lucifer yellow (LY, potassium salt, MW = 522 Da; Thermo Fisher Scientific, Switzerland) dye mix (50 µg/mL) was added. The media in the basolateral compartment was changed with 3 mL PBS. The plates were incubated in the dark at 20°C, and 100 µl samples were taken from the basolateral compartment after 20, 30, 60, 90 and 120 min and added to conventional 96-well plates without inserts. Additionally, 100 µl samples were taken from the apical compartment after 20 and 120 min. When only sampling from the basolateral compartment, the volume was corrected by adding 100 µl PBS. Fluorescence was measured immediately after sampling using a microplate reader (VLB000 Varioskan™ LUX mulitimode) with excitation/emission wavelengths of 450/520 nm. Fluoresence readings were recorded as relative fluorescent units (RFUs) based on a single read and converted to the concentration of passed LY based on a separately made standard curve. Background fluorescence from wells without cells (blank) was subtracted from both control and stimulated cells. After the last sampling of LY, the LY dye mix was removed, and the cells washed twice with 1 mL PBS before cells were sampled for qPCR. The apical compartment of the last three replicates were washed with 1 mL PBS and then incubated with 60 µg/mL 3 mL LPS for 6 hours. After this period, the LY sampling protocol was conducted as previously described, followed by cell collection for qPCR analysis (**Supplementary Figure 2**). Apparent permeability (P_app_) for LY was calculated according to Hubatsch et al., 2007 (protocol 2) using Equation 1.


(1) $$Papp\;=\left(\frac{dQ}{dt}\right)\;x\;\left(\frac{1}{AC_{0}}\right)$$

where $\left(\frac{dQ}{dt}\right)$ is the quantity of LY on the basolateral side as a function of time (µmol s^-1^), C_0_ the initial concentration of LY on the apical side (µmol/mL), and A the surface area of exposed RTgutGC monolayer on the transwell inserts (4.520 cm^2^).

### 2.6 Quantitative real time PCR

After conducting the LY translocation assay, the cells were harvested for qPCR analysis at day 21 (**Supplementary Figure 2**). Briefly, after rinsing with PBS, 600 µl TRI Reagent solution (Direct-zol RNA Miniprep Plus kit, Zymo Research Corporation) was added directly to the RTgutGC monolayer. The cells were detached and lysed by repeated pipetting and scraping of the insert bottom 8–10 times. The resulting cell homogenates were transferred to 2 mL cryogenic tubes (479-1220, VWR), placed on ice, and then stored at -80°C until further processing. Total RNA was subsequently extracted using the Direct-zol RNA Mini Plus kit (Zymo Research Corporation) according to the manufacturer’s protocol, which included DNase I treatment step to remove any residual genomic DNA that could interfere with the PCR reactions. The RNA was eluted in 50 µl of RNase-free water. To ensure complete elution, the eluate was reapplied to the column and centrifuged at 12 000 x g. RNA purity and concentration were measured using a NanoDrop ND-1000 spectrometer (ThermoFisher Scientific), with all samples showing a 260:280 greater than 1.9. RNA integrity was confirmed using an Aligent 2100 Bioanalyzer with a DNA Nano Chip (Agilent Technologies, CA, USA) yielding a RIN value of greater than 9. Total RNA was stored at -80°C until further use. First-strand complementary DNA (cDNA) was synthesized from 1.0 µg of total RNA using LunaScript RT SuperMix kit (New England Biosystems). Negative controls were performed in parallel by excluding either RNA or the enzyme.

The 13 chosen target genes had key roles in immune signaling and inflammatory responses; *il6* (interleukin-6, IL-6), *il8* (interleukin-8, IL-8), *il1b* (interleukin-1β, IL-1β), *tnfa* (tumor necrosis factor-alpha, TNF-a), *myd88* (myeloid differentiation primary response 88, MyD88), barrier function and integrity; *cdh1* (E-cadherin, CDH1), *cldn3* (claudin-3), *cldn12* (claudin-12), *zo-1* (zonula occludens-1, ZO-1) and metabolic processes; *tgfb* (transforming growth factor-beta, TGF-β), *pcna* (proliferating cell nuclear antigen, PCNA), *ialp* (intestinal alkaline phosphatase, IALP) and *slc10a2* (apical sodium-dependent bile acid transporter, SLC10A2). Primers for these genes were designed using NCBI’s Primer-BLAST or sourced from existing literature (**Supplementary Table 3**). All primers were ordered from Invitrogen by Thermo Fisher Scientific in a hydrophilized state. The optimal annealing temperatures for each gene’s primer pair (forward and reverse) were tested on a pool of cDNA from all samples using gradient PCR method with temperatures ranging from 54.0 – 62.0°C. Final annealing temperatures were chosen based on amplification curves and melting peak analyses. PCR efficiency was calculated for each gene assay via two-fold serial dilutions of pooled cDNA, using LinReg PCR software (54, 55). Gene expression (quantified by Cq values) was analyzed on a LightCycler 96 system (Roche Diagnostics, Basel, Switzerland) using LightCycler 480 SYBR Green I Master Mix. Each 20 µL PCR reaction contained 3 µL PCR-grade water, 2 µL of forward and reverse primers (10 µM), 10 µL SYBR Green I Master Mix, and 5 µL cDNA template diluted at either 1:10 (*il8, il1b, tnfa, myd88, tgfb, pcna, ialp, cdh1, cldn3, cldn12* and *zo-1*) or 1:5 (*il6* and s*lc10a2*). Negative controls (without cDNA) and no-RT controls were included. After detecting a low amount of gDNA in the samples, the samples were cleaned using RNA Clean & Concentrator-5 (Biosite-R1013, Zymo Research). The qPCR protocol consisted of an enzyme activation step at 95°C for 10 minutes, followed by 45 amplification cycles: denaturation at 95°C for 15 seconds, annealing at 55-58°C (temperature optimized per gene, see **Supplementary Table 3**) for 15 seconds, and elongation at 72°C for 15 seconds. Target gene expression was normalized to the geometric mean of actin beta (*actb*) and elongation factor 1α (*EF1a*), selected for their stable intra- and interspecific expression as confirmed using the geNORM function (56) in qbase. Mean normalized fold change was calculated from raw Cq values by relative quantification using Equation 2 (57).


(2) $$Fold\;change=2^{-\text{ΔΔCq}}$$

### 2.7 Statistical analysis

All statistical analysis was performed in R Studio (R version 2024.04.1, ^©^2009-2024, RStudio, PCB). Normal distribution was visualized using Q-Q plot and Shapiro-Wilks normality test, and homogeneity of variance was tested using Levene’s test. Data were analyzed using two-way ANOVA with (LPS) challenge and treatment as class variables followed by Dunnett’s multiple comparisons test within each challenge group (before and after LPS challenge) (**Supplementary Tables 4** and **5**). The Dunnett’s test was chosen to compare multiple treatments to its respective control group while controlling the overall Type I error rate (58). Data were calculated as mean ± SEM with 3 or 4 technical well or insert replicates (given in the figure legends). Asteriks denote treatment groups that are statistically different from the control (*** *p* < 0.001, ** *p* < 0.01, * *p* < 0.05), whereas trends are denoted by “.” (0.05 ≤ *p* < 0.10).

## 3 Results

### 3.1 Selection of dosages for cell trial (cell viability)

**Figure 2:**
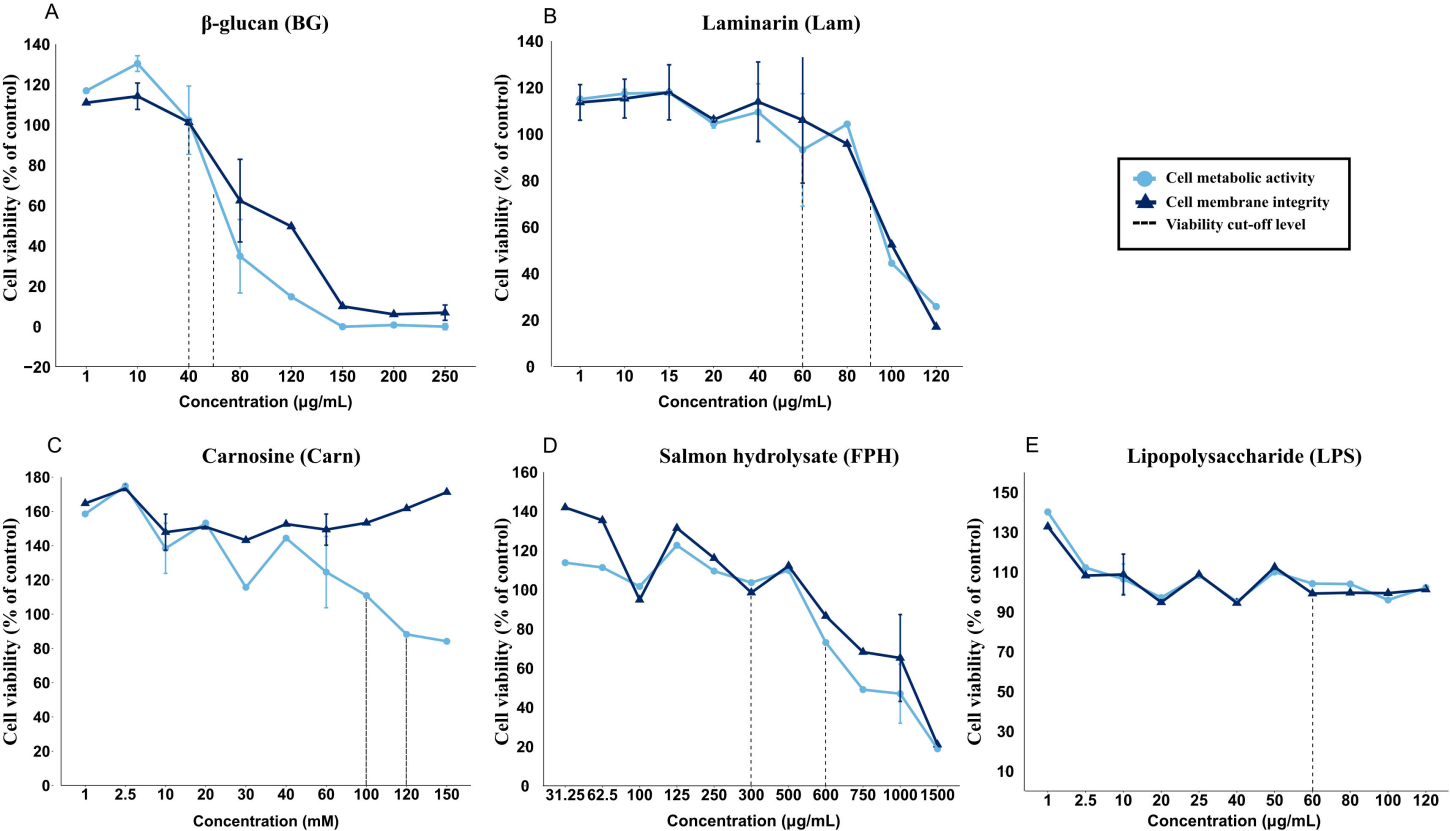
Viability of RTgutGC cells exposed to various concentrations of **(A)** β-glucan (BG, µg/mL), **(B)** laminarin (Lam, µg/mL), **(C)** carnosine (Carn, mM), **(D)** salmon hydrolysate (FPH, µg/mL) and **(E)** lipopolysaccharide (LPS, µg/mL). Stippled line represents approximately 80 % and 100 % cell viability compared to the control cells. The data represent mean ± SEM of two independent experiments with 4 technical well-replicates for overlapping concentrations, whereas for concentrations only evaluated in one of the experiments, single mean values of 4 technical well-replicates are plotted.

For most functional feed ingredients, the cell viability measured by a quantitative measure of cell metabolic activity (AB assay) and cell membrane integrity (CFDA-AM assay) decreased with increasing inclusion levels. Except for carnosine, where the cell metabolic activity decreased while the cell membrane integrity did not – these two measurements followed each other. For further testing, two levels of inclusion were chosen, the highest level of an ingredient giving 100 % viability, and the second level giving 80 % viability. This gave the following working concentrations; β-glucan with 40 µg/mL and 60 µg/mL (BG40 and BG60) (**Figure 2A**), laminarin with 60 µg/mL and 90 µg/mL (Lam60 and Lam90) (**Figure 2B**), L-Carnosine with 100 mM and 120 mM (Carn100 and Carn120) (**Figure 2C**) and salmon hydrolysate with 300 µg/mL and 600 µg/mL (FPH300 and FPH600) (**Figure 2D**). LPS did not affect viability (**Figure 2E**) and a concentration of 60 µg/mL were chosen based on literature data (48). All test concentrations are shown in **Supplementary Table 2**.

### 3.2 TEER and Lucifer yellow translocation

#### 3.2.1 Glucans

**Figure 3:**
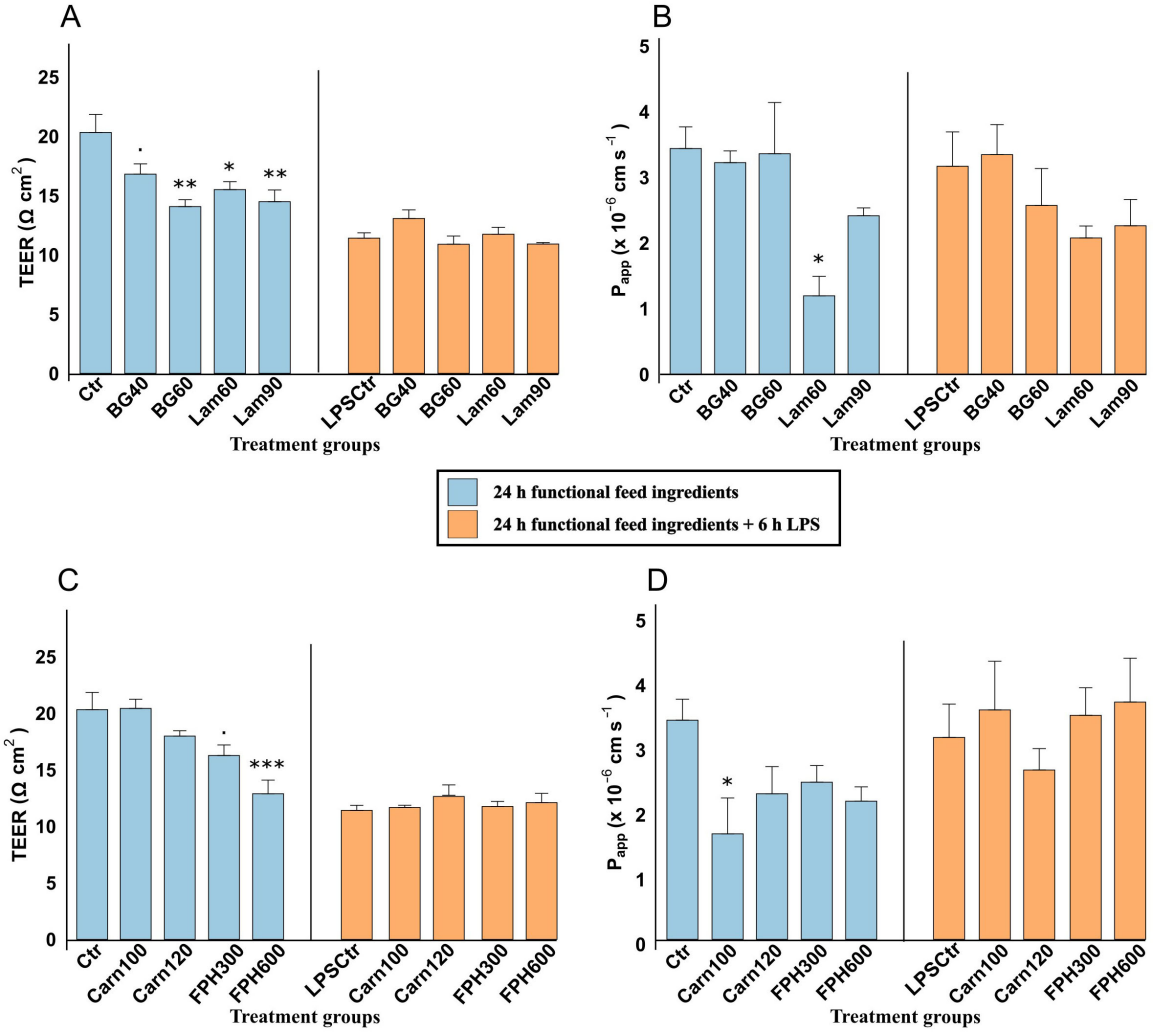
Transepithelial electrical resistance (TEER, Ω cm^2^) and Lucifer yellow apparent permeability (P_app_ x 10^-6^ cm s^-1^) of RTgutGC cells exposed to 40- and 60 µg/mL β-glucan (BG) and 60- and 90 µg/mL laminarin (Lam) **(A, B)**, and 100- and 120-mM carnosine (Carn) and 300- and 600 µg/mL salmon hydrolysate (FPH) and **(C, D)**. Blue color shows TEER and P_app_ after being exposed to functional feed ingredients for 24 hours, and orange color shows TEER and P_app_ after being pretreated with functional feed ingredients for 24 hours, before being challenged with LPS for 6 hours. Data represent mean ± SEM of 4 (TEER) and 3 (P_app_) technical well-replicates per treatment. Asterisks denote treatment groups that are statistically different from the control (*** *p* < 0.001, ** *p* < 0.01, * *p* < 0.05), whereas trends are denoted by “.” (0.05 ≤ *p* < 0.10).

Exposure to β-glucan for 24 hours appeared to result in a dose-dependent reduction in TEER, decreasing from 20.32 ± 1.52 Ω cm^2^ in the control group to 16.80 ± 0.87 Ω cm^2^ in BG40 (0.05 ≤ *p* < 0.10) and 14.07 ± 0.53 Ω cm^2^ in BG60 (*p* < 0.01) (**Figure 3A**). Similar reduction was caused by Lam60 (*p* < 0.05) and Lam90 (*p* < 0.01). The 6-hour LPS challenge caused a general reduction in TEER (11.40 ± 0.45 Ω cm^2^ in the LPS-control group) (*p* < 0.001) (**Supplementary Table 4**). The pretreatment with glucans did not impact this reduction.

The apparent permeability (P_app_) of lucifer yellow of cells exposed to both concentrations of β-glucan for 24 h was 3.29 ± 0.36 x 10^-6^ cm s^-1^ (**Figure 3B**). Both laminarin concentrations appeared to reduce the apparent permeability from 3.44 ± 0.33 x 10^-6^ cm s^-1^ in control to 1.20 ± 0.30 x 10^-6^ cm s^-1^ in Lam60 and 2.42 ± 0.49 x 10^-6^ cm s^-1^ in Lam90, but the difference was only significant with the lowest concentration (*p* < 0.05). The addition of LPS did not significantly impact the permeability of Lucifer yellow (**Supplementary Table 4**), and there were no significant differences in the cells exposed to any glucans after the challenge.

#### 3.2.2 Bioactive peptides

A 24-hour exposure to increasing concentrations of salmon hydrolysate reduced the TEER in a dose dependent manner from 20.32 ± 1.52 Ω cm^2^ in the control group to 16.24 ± 0.93 Ω cm^2^ in FPH300 (*p* = 0.05) and 12.88 ± 1.20 Ω cm^2^ in FPH600 (*p* < 0.001) (**Figure 3C**). None of the exposure concentrations of carnosine (20.43 ± 0.81 Ω cm^2^ and 17.98 ± 0.47 Ω cm^2^ in Carn100 and Carn120, respectively) had a significant effect on TEER compared to the control. Exposure of cells to 6 hours of LPS after 24-hour pretreatment with bioactive peptides, had no further effect on TEER rather than the general reduction caused by LPS itself as noted above (**Supplementary Table 5**).

The P_app_ values of cells exposed to salmon hydrolysate averaged 2.32 ± 0.17 x 10^-6^ cm s^-1^ which was not significantly lower than control (**Figure 3D**). The P_app_ values of the carnosine groups inversely proportionally followed the TEER values, with lowest P_app_ in Carn100 (1.66 ± 0.40 x 10^-6^ cm s^-1^) which significantly differed from the control group (*p* < 0.05). After the addition of LPS, there were no differences between the bioactive peptides.

### 3.3 Gene expression

#### 3.3.1 Glucans

**Figure 4:**
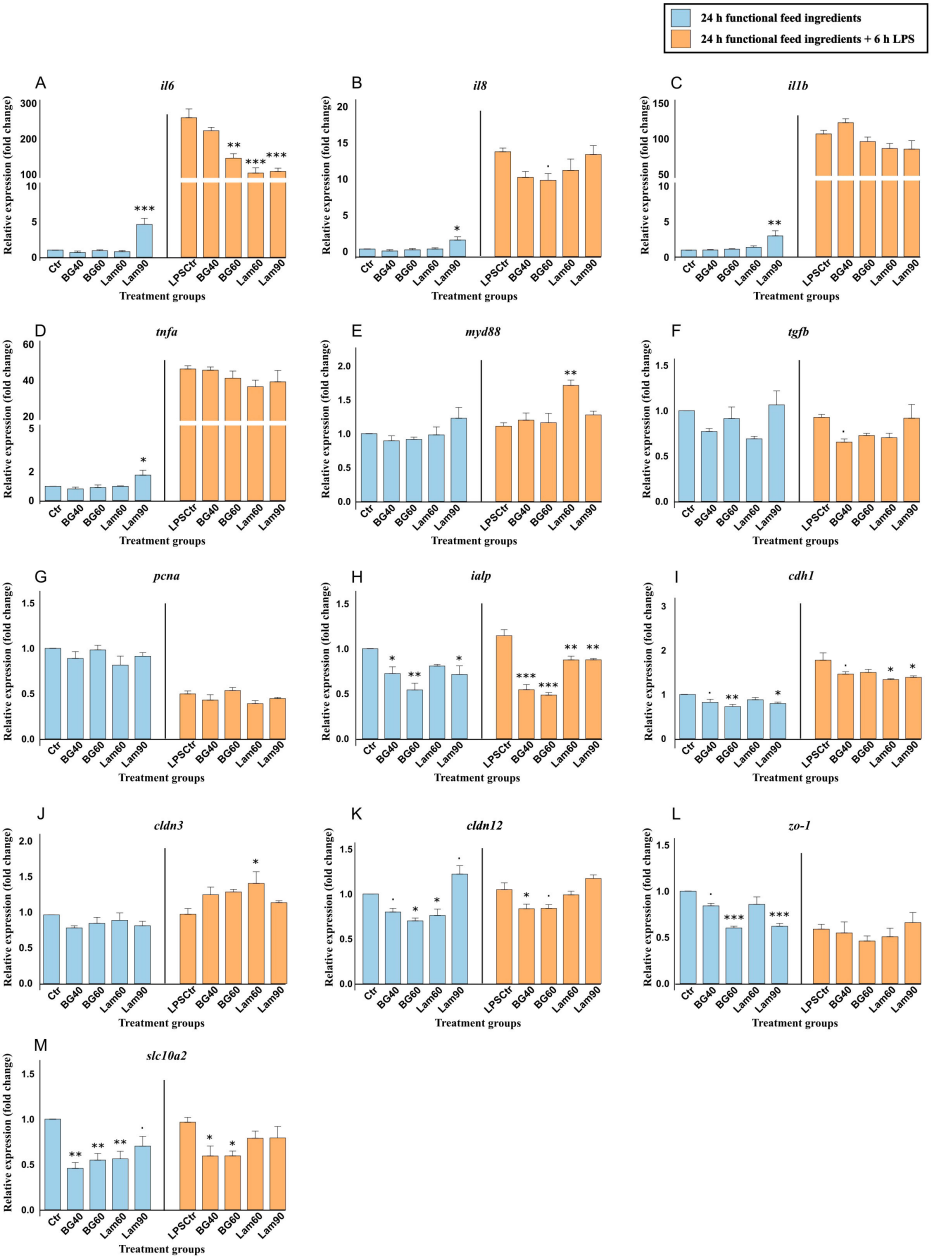
Gene expression of important immune **(A-F)**, barrier **(I-L)**, and metabolic **(G, H, M)** genes in RTgutGC cells exposed to 40- and 60 µg/mL beta-glucan (BG) and 60- and 90 µg/mL laminarin (Lam). Blue color shows gene expression after being exposed to functional feed ingredients for 24 hours and orange color shows gene expression after being pretreated with functional feed ingredients for 24 hours before being challenged with 6 h LPS. Data are expressed relative to the control cell level without LPS and represent mean ± SEM of 3 technical well replicates each. Asterisks denote treatment groups statistically significant to its respective control group (***p < 0.001, **p < 0.01, *p < 0.05), whereas trends are denoted by “.” (0.05 ≤ *p* < 0.10).

A 24-hour exposure of Lam90 led to marked increase mRNA of several pro-inflammatory genes such as interleukin-6 (*il6*) (*p* < 0.001) (**Figure 4A**), interleukin-8 (*il8*) (*p* < 0.05) (**Figure 4B**), interleukin-1β (*il1b*) (*p* < 0.01) (**Figure 4C**) and tumor necrosis factor alpha (*tnfa*) (*p* < 0.05) (**Figure 4D**) compared to the control. There were no difference between treatments in expression of transforming growth factor beta (*tgfb*) (**Figure 4F**). Both β-glucan and laminarin had a suppressing effect on the expression of intestinal alkaline phosphatase (*ialp*) (**Figure 4H**) involved in lipid metabolism and gut health, as well as sodium/taurocholate cotransporting polypeptide (*slc10a2*) (**Figure 4M**), involved in bile transport and metabolism. There was also, with some exceptions, a general suppression of the genes related to maintaining epithelial or cellular barrier integrity such as E-cadherin (*cdh1*) (**Figure 4I**), claudin-12 (*cldn12*) (**Figure 4K**) and zonula occludens-1 (*zo-1*) (**Figure 4L**). Interestingly, the highest dose of laminarin (Lam90) tended to increase the expression of *cldn12* (0.05 ≤ *p* < 0.10).

Exposing the cells to 6 hours LPS led to manyfold increase in the expression of mRNA coding for the pro-inflammatory genes *il6* (**Figure 4A**), *il8* (**Figure 4B**), *il1b* (**Figure 4C**) and *tnfa* (**Figure 4D**) (*p* < 0.001) (**Supplementary Table 4**). LPS also caused a decrease in proliferating cell nuclear antigen (*pcna*) and *zo-1* (*p* < 0.001), and an increase in *cdh1* (*p* < 0.001). Within the LPS challenged group, BG40, Lam60 and Lam90 reduced the expression of *il6*, but they had little effect on the other inflammatory genes except for Lam60 that increased the expression of myeloid differentiation primary response 88 (*myd88*) (*p* < 0.01) (**Figure 4E**). Interestingly, Lam60 also increased the expression of *cldn3* (*p* < 0.05) (**Figure 4J**) involved in maintaining barrier integrity, and while expression of *cldn12* (**Figure 4K**) was reduced by β-glucan, there were no difference between control and the laminarin groups. On the contrary, both Lam60 and Lam90 significantly reduced the expression *cdh1* (*p* < 0.05) (**Figure 4I**) while BG40 and BG60 did not. Pretreatment with β-glucan before the LPS challenge led to downregulation in expression of sodium/taurocholate cotransporting polypeptide (*slc10a2*) (**Figure 4M**), but laminarin did not have this effect.

#### 3.3.2 Bioactive peptides

**Figure 5:**
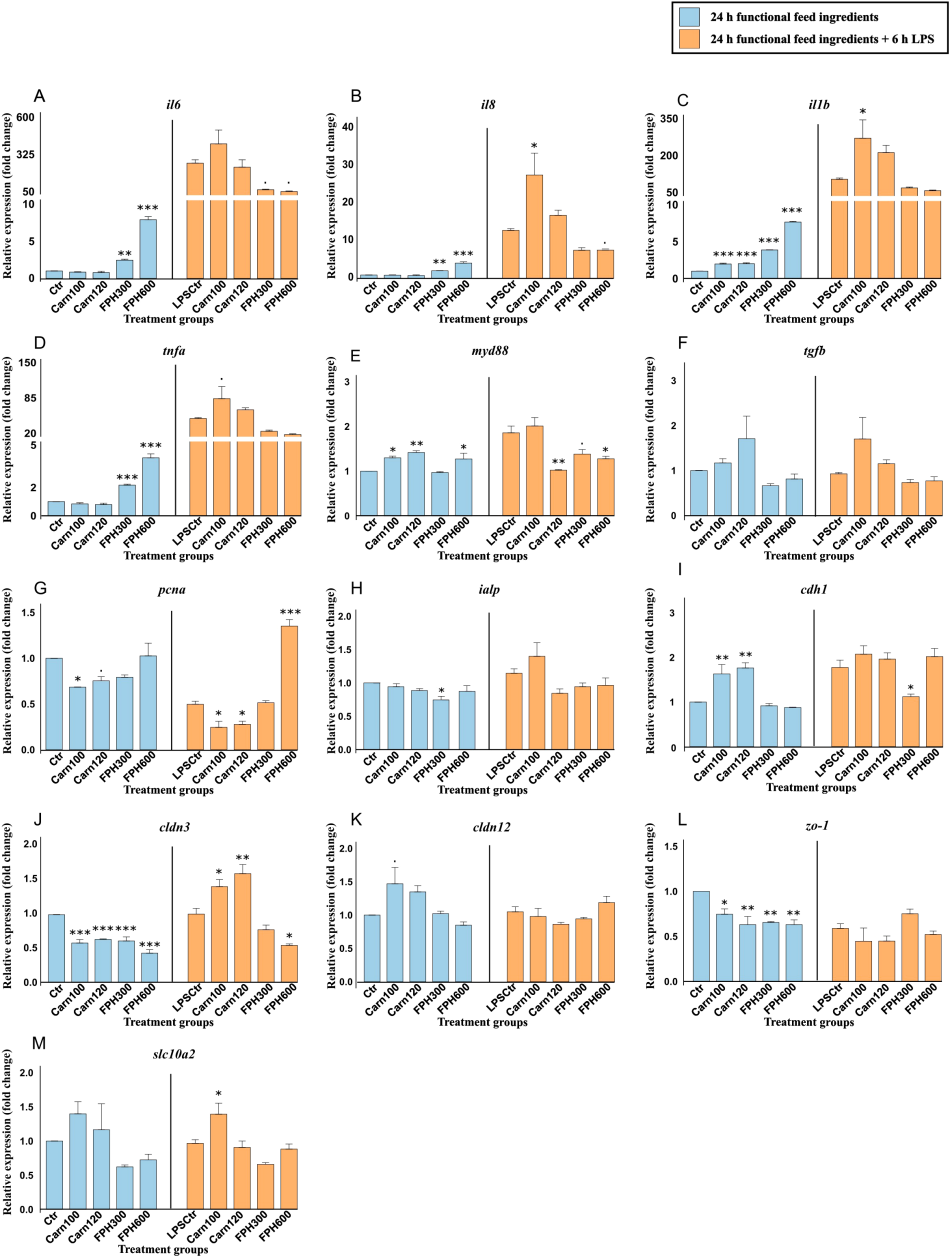
Gene expression of important immune, barrier, and metabolic genes in RTgutGC cells exposed to 100- and 120-mM carnosine (Carn) and 300- and 600 µg/mL salmon hydrolysate (FPH). Blue color shows gene expression after being exposed to functional feed ingredients for 24 hours and orange color shows gene expression after being pretreated with functional feed ingredients for 24 hours before being challenged with 6 h LPS. Data are expressed relative to the control cell level without LPS and represent mean ± SEM of 3 technical well replicates each. Asterisks denote treatment groups statistically significant to its respective control group (*** *p* < 0.001, ** *p* < 0.01, * *p* < 0.05), whereas trends are denoted by “.” (0.05 ≤ *p* < 0.10).

A 24-hour exposure to salmon hydrolysate significantly increased the expression of the pro-inflammatory *il6* (**Figure 5A**), *il8* (**Figure 5B**), *tnfa* (**Figure 5D**) compared to the control. Interleukin-1β (*il1b*) (**Figure 5C**) was also increased by both carnosine concentrations (*p* < 0.001). Another inflammatory gene, *myd88* (**Figure 5E**) was increased by Carn100, Carn120 and FPH600, but not FPH300. On the contrary, the expression of *ialp* was only significantly reduced by FPH300 (*p* < 0.05) (**Figure 5H**) and not the other treatments. Another metabolic gene, *pcna*, more specifically linked to cell proliferation and repair, was downregulated by Carn100 (*p* < 0.05) and tended to be downregulated by Carn120 (0.05 ≤ *p* < 0.10) (**Figure 5G**). The genes related to barrier integrity, such as *cldn3* and *zo-1* was markedly suppressed by both carnosine and salmon hydrolysate (**Figures 5J, K**, respectively). E-cadherin, however, was significantly increased by both concentrations of carnosine (**Figure 5I**).

As mentioned above, exposing the cells to 6 hours of LPS led to a marked increase in several of the pro-inflammatory genes (**Supplementary Table 5**). Within the LPS challenged group, Carn100 increased the expression of *il8* (*p* < 0.05) (**Figure 5B**), *il1b* (*p* < 0.05) (**Figure 5C**) and tended to increase the expression of *tnfa* (0.05 ≤ *p* < 0.10) (**Figure 5D**). Expression of *myd88* (**Figure 5E**) was significantly reduced by Carn120 and FPH600 and tended to be reduced by Carn300. While being downregulated by both carnosine concentrations, the *pcna* was markedly upregulated by FPH600 (**Figure 5G**) (*p* < 0.001). The regulation of *slc10a2* (**Figure 5M**) was only affected by Carn100 which increased the expression (*p* < 0.05) compared to the control exposed to LPS. Among the genes important in barrier integrity, FPH300 reduced the expression of *cdh1* (*p* < 0.05) (**Figure 5I**). FPH600 reduced the expression of *cldn3* whereas Carn100 and Carn120 significantly increased the expression compared to control exposed to LPS (**Figure 5J**).

## 4 Discussion

Finding sustainable functional feed ingredients that enhance the intestinal epithelial health of farmed salmonids could be an important solution to improve fish resilience to diseases, robustness and general fish health. These ingredients include β-glucans, which can strengthen immunity, and bioactive peptides, which offer immunomodulatory and antimicrobial effects (27, 32). In the present paper, it was investigated how exposure to two different concentrations- of glucans; β-glucan from baker’s yeast (*S. cerevisiae*) and laminarin from *L. hyperborea*, and bioactive peptides; carnosine and salmon hydrolysate for 24 hours impacted the barrier function and gene expression of the RTgutGC cells under normal conditions and after exposure to 6-hours LPS induced inflammation.

### 4.1 Effect of functional feed ingredients and LPS on RTgutGC cell viability

Based on the cell viability assay, two concentrations of each functional feed ingredient were selected. The first was a concentration that maintained 100 % viability, ensuring no adverse effects on cell functionality. This concentration could be safely incorporated into diet formulations during challenging farming conditions. The second concentration resulted in approximately 80 % viability, balancing minimal toxicity with a sufficient response to evaluate the ingredient’s effects. The assay revealed that increasing concentrations of all functional feed ingredients lowered the cell metabolic activity, while the cell membrane integrity was lowered by all, except carnosine. This suggests that while increasing concentrations of carnosine reduces metabolic activity, it is not directly cytotoxic. The observed effect may be due to a combination of carnosine’s antioxidant properties, which can modulate mitochondrial function and reduce oxidative stress without inducing membrane damage (59, 60), and the fact that high concentrations of carnosine are found in the gut, brain and muscle of fish and other vertebrates (61). In contrast, the other ingredients that reduced both metabolic activity and membrane integrity likely triggered apoptotic or necrotic pathways (62). However, the specific mechanisms were not the focus of this study, as our main emphasis was on immune- and barrier regulation. The viability of the RTgutGC cells was not affected by any of the tested LPS concentrations, consistent with previous studies (47, 48). While fish are generally considered less responsive to LPS than mammals due to the absence of the LPS-specific receptor TLR4 and co-stimulatory molecules, the chosen concentration has been shown to induce significant immunomodulatory effects (43, 47, 63, 64). Moreover, crude LPS preparations often contain contaminants such as peptidoglycans, nucleic acids and lipoproteins which are potent inducers of pro-inflammatory cytokine gene expression despite having no apparent impact on cell viability (65).

### 4.2 Effect of functional feed ingredients on barrier integrity and immune regulation of RTgutGC cells

β-glucans are known to be very potent immune stimulants in aquaculture, with beneficial effects on growth, immune modulation and disease resistance (27, 66, 67). In the present trial, the highest concentration of laminarin (90 µg/mL) from *L. hyperborea* upregulated all measured pro-inflammatory cytokines, including *il6*, *il8*, *il1b* and *tnfa* compared to the control. This was not seen with the lower concentration of laminarin or any of the concentrations of β-glucan from *S. cerevisiae*, indicating a higher immunomodulating potency in laminarin. In mammals, the more structurally complex β-glucans, such as β-glucan from *S. cerevisiae* generally exhibit stronger immunomodulating effects compared to the lesser structurally complex β-glucans, such as laminarin (68, 69). This is because their structure usually interact more effectively with pattern recognition receptors (PRRs) such as Dectin-1-like receptors and Toll-like receptors (TLRs) expressed on immune cells (68–70). While the exact mechanism in fish is less well-defined, studies have suggested that fish possess analogous PPRs, such as C-type lectins, which may recognize β-glucans and modulate immune responses in gut epithelial cells (71). This interaction can enhance the expression of immune-related genes and promote the secretion of cytokines. In the current experiment, the higher potency of response by laminarin on the pro-inflammatory *il6*, *il8*, *il1b* and *tnfa*, than conventional β-glucan was unexpected and needs further examination. The result nevertheless does support notions that that the immune modulation of β-glucans is highly variable depending on a lot of factors like structure, solubility and exposure time (26, 27, 69, 70, 72, 73).

Bioactive peptides have also shown to have different antioxidant and immunomodulatory properties depending on the source and structure (32, 41, 74, 75). In the present work, salmon hydrolysate increased the expression of all pro-inflammatory cytokines in a dose dependent manner compared to the control, while carnosine was found to increase the expression of *il1b*. Although the concentration of carnosine is much higher than that of salmon hydrolysate (100 mM carnosine = 22 626 µg/mL), RTgutGC cells seem to be much more responsive to salmon hydrolysate. This is likely because of the natural occurrence of carnosine in the fish gut (61), and because of the complex composition of salmon hydrolysate. Unlike carnosine, which only consists of the dipeptide β-alanine and histidine, the diverse range of bioactive peptides in addition to a broader amino acid composition in salmon hydrolysate may have synergistic effects that provides enhanced immune signaling (32, 76). Moreover, it is also likely that hydrolysates exhibit higher bioavailability due to their smaller peptide fractions, facilitating more efficient cellular uptake and utilization compared to carnosine (32, 77). Fish protein hydrolysate has previously been shown to enhance the expression of pro-inflammatory cytokines in mice while still maintaining the intestinal homeostasis (78). Similar results were found in Mallet et al., 2014, where the increased expression of pro-inflammatory cytokines after feeding mice with shark-derived protein hydrolysate resulted in reduced inflammation in response to *E. coli* infection (79). While the effect of fish protein hydrolysate on the gut barrier- and immune function of fish is relatively scarce, Sandbakken et al., 2024, found that salmon hydrolysate downregulated the expression of immune related genes in Atlantic salmon (*Salmo salar*) intestinal segments compared to in fish fed fish meal. Nonetheless, controlled stimulation of immune cells or in this case, fish gut epithelial cells, to produce pro-inflammatory cytokines may support a more effective immune response during exposure to pathogens (80).

In the present trial, both β-glucan from *S. cerevisiae*, laminarin from *L. hyperborea* and salmon hydrolysate reduced the TEER in a dose dependent manner compared to the control, indicating a decrease in epithelial barrier integrity (81). This reduction of TEER in the intestine, could facilitate increased antigen influx and further trigger mucosal immune responses possibly leading to excessive inflammation (82). For the highest concentrations of β-glucan and laminarin, the reduction in TEER was accompanied by a decreased expression in adherence junction-related E-cadherin (*cdh1*) indicating weakened adherent junctions, and *zo-1*, which is essential for tight junction structure. Furthermore, they reduced the expression of intestinal alkaline phosphatase (*ialp*), which is an important brush border enzyme that destroys bacterial LPS (83). Addition of both salmon hydrolysate and carnosine also reduced the expression of the important barrier genes *cldn3* and *zo-1*, however, salmon hydrolysate did not affect expression of *cdh1* while carnosine, in fact, increased the expression. Interestingly, the lowest concentration of carnosine (100 mM) also tended to increase the expression of *cldn12* involved in ion permeability regulation (84). As such, this concentration of carnosine may be promoting tight junction remodeling, potentially altering epithelial barrier permeability.

Tight junction selectivity and permeability toward ions are regulated by the specific types of claudins present in the junctional pores (85). In contrast, the apparent permeability (P_app_) to larger molecules is primarily influenced by pore size, which can be modulated by the activity of myosin light chain kinase (MLCK) (86). Importantly, permeability via the pore and leak pathways can be regulated independently (86–89). This is seen by the effects observed with the low-dose laminarin, where both TEER and P_app_ of Lucifer yellow decreased. A reduction in TEER, indicating increased ion permeability, may reflect a shift in claudin isoform expression within tight junctions (85). On the other hand, a decrease in P_app,_ reflecting reduced permeability to larger molecules, could result from increased expression of occludin and/or decreased MLCK activity (86, 87). Such dual regulation, where both TEER and P_app_ are reduced has also been observed in the proximal intestine of Atlantic salmon (*S. salar*) (11). Further evidence of selective regulation of the leak pathway is provided by the low-dose carnosine treatment, which decreased P_app_ without affecting TEER (44, 82).The lowest concentrations of laminarin and carnosine significantly reduced P_app_, suggesting formation of smaller pores that limit the passage of larger, uncharged molecules. These findings indicate that compensatory mechanisms are activated that enhance the paracellular barrier function by tightening junctions or selectively restricting permeability to certain solutes. Tight junction regulation by feed additives in fish are limited and warrants further investigation, however it has been seen that supplementation of laminarin in appropriate concentrations improves the gut health and digestive function of juvenile spotted seabass (*Lateolabrax maculatus*) (31).

### 4.3 Effect of LPS-induced inflammation on barrier integrity and immune regulation of RTgutGC cells after pretreatment with functional feed ingredients

The 6-hour LPS exposure caused a combination of inflammatory response, barrier dysfunction and compensatory mechanisms. More specifically, LPS triggered an inflammatory response leading to upregulation of genes coding for cytokines that are key mediators of inflammation (*il6*, *il8*, *tnfa* and *il1b*), a result that is in accordance with other studies (43, 48, 90). While these cytokines contribute to the cells’ immune defense against bacteria, their overproduction can cause local inflammation and tissue stress leading to cellular damage (91). This in turn weaken the epithelial barrier as reflected in the decreased TEER and disruptions of tight junction proteins like zonula occludens-1 (ZO-1) on a molecular level in the present study. Cytokines can induce the downregulation or removal of tight junction proteins, or activate myosin light kinase, which contracts the actin cytoskeleton. This strain on the tight junction can create cracks in the barrier, further compromising epithelial integrity (92). LPS also caused a decrease in the gene coding for proliferating cell nuclear antigen (*pcna*), an important marker of cell proliferation (93). This could indicate that the cells shift cellular resources away from proliferation toward managing damage. Exposure to LPS led to an increase in E-cadherin (*cdh1*) expression, a key protein involved in maintaining epithelial cell adhesion. This aligns with the findings of Wang et al. (2019), who observed a similar response in RTgutGC cells exposed to LPS. In contrast, studies in mammals generally report a downregulation of E-cadherin following LPS exposure (94, 95). This species-specific difference may be attributed to the unique challenges faced by fish, whose intestines are continuously exposed to waterborne pathogens, including LPS from abundant aquatic Gram-negative bacteria. While mammals rely more on their immune system to clear infections, fish may prioritize maintaining epithelial integrity as a frontline defense (4). Upregulating E-cadherin could serve as a protective strategy to reinforce the intestinal barrier and counteract the disruptive effects of LPS on cell adhesion.

Interestingly, pretreatment with the high-dose of β-glucan and both the concentrations of laminarin reduced the expression of *il6* compared to the LPS-control. Pretreatment with salmon hydrolysate also showed the same trend. This could support the idea that these functional feed ingredients had priming effects by which they enhanced the immune system’s readiness to respond to pathogens while also preserving excessive inflammation as supported by other studies (40, 69). For instance, lentinan, a β-glucan extracted from the mushroom *Lenitula edodens* reduced the expression of genes associated with LPS-induced inflammation in the head kidney of rainbow trout (*O. mykiss*) (80). The anti-inflammatory properties of β-glucan from various sources was also revealed in an LPS-induced RAW 264.7 cell model (96). In another study the LPS-induced RAW 264.7 cell model was used after pretreatment with salmon hydrolysate, which resulted in reduced expression of pro-inflammatory cytokines (97). The potential beneficial effect of β-glucan and laminarin on LPS-induced inflammation is further supported by downregulation in *ialp*. This suggests that when these ingredients are effective, the cells may not need to upregulate *ialp* as much for the detoxification of LPS, implying a reduction in the inflammatory burden (98). The lowest concentration of carnosine, on the other hand, increased the expression of the pro-inflammatory cytokines *il8* and *il1b* and showed a tendency to increase the expression of *tnfa*. This is rather surprising as other studies have found that carnosine protects against the oxidative damage induced by LPS and H_2_O_2_ (36, 99–101).

The lowest concentration of laminarin significantly upregulated the expression of the myeloid differentiation primary response protein (*myd88*) in response to LPS stimulation, compared to the LPS-control group. MyD88, an adaptor protein, is critical for initiating downstream signaling cascades such as NF-κB activation. This pathway facilitates a robust cellular response to LPS by promoting the release of pro- and anti-inflammatory cytokines, thereby modulating the immune response to bacterial challenges (102). The same treatment also increased the *cldn3* expression suggesting a potential protective or regulatory role in maintaining epithelial barrier integrity during inflammation (82). This could be related to laminarins’ antioxidant capacity in the fish gut possibly reducing the damage by reactive oxygen species (ROS) produced by LPS exposure, as reported by Qiu et al. (2023). The highest concentration of carnosine and both concentrations of salmon hydrolysate, on the other hand, downregulated *myd88* which could indicate an immune-modulatory effect, possibly to ensure a more controlled immune response after the inflammatory activation caused by LPS (103). Both concentrations of carnosine also upregulated *cldn3*, and the highest concentration of salmon hydrolysate counteracted the effect of LPS on cell proliferation by upregulating *pcna*. This could be beneficial for tissue recovery, especially where the barrier function may be compromised after LPS exposure (104).

## 5 Conclusion

Functional feed ingredients hold strong potential to enhance immune function and barrier integrity in aquaculture. This study confirms the RTgutGC cell line as a valuable *in vitro* model for screening such ingredients, though *in vivo* validation remains essential due to the complexity of gut-immune interactions. The results highlight the importance of ingredient type, concentration, and origin in determining their biological activity and immunomodulatory effects. Notably, high-dose of laminarin and both concentrations of salmon hydrolysate, triggered acute immune responses, which could be beneficial for immune priming before stress events. However, balancing immune activation with barrier integrity, is crucial to avoid adverse effects. LPS exposure in the RTgutGC cells elicited a combination of inflammatory responses, barrier dysfunction, and compensatory mechanisms. Pretreatment with β-glucan, laminarin, and salmon hydrolysate appeared to reduce the inflammatory response to LPS and high-dose salmon hydrolysate supported tissue recovery. Priming the fish’s immune system in aquaculture can increase their ability to develop a faster and stronger response to pathogens, reducing the risk of disease outbreaks and improving survival rates. Ultimately, this may enhance both economic and environmental sustainability in the industry.

## Data Availability

The datasets presented in this study can be found in online repositories. The names of the repository/repositories and accession number(s) can be found in the article/**Supplementary Material**.
