# Supplementary material for: Functional feed ingredients modulate the immune response of RTgutGC cells to LPS-induced inflammation
Source: Front Immunol. 2025 Jun 18;16:1616076. doi: 10.3389/fimmu.2025.1616076 (PMC12219272; doi:10.3389/fimmu.2025.1616076)
Supplement: Supplementary file 1 [file DataSheet1.pdf]

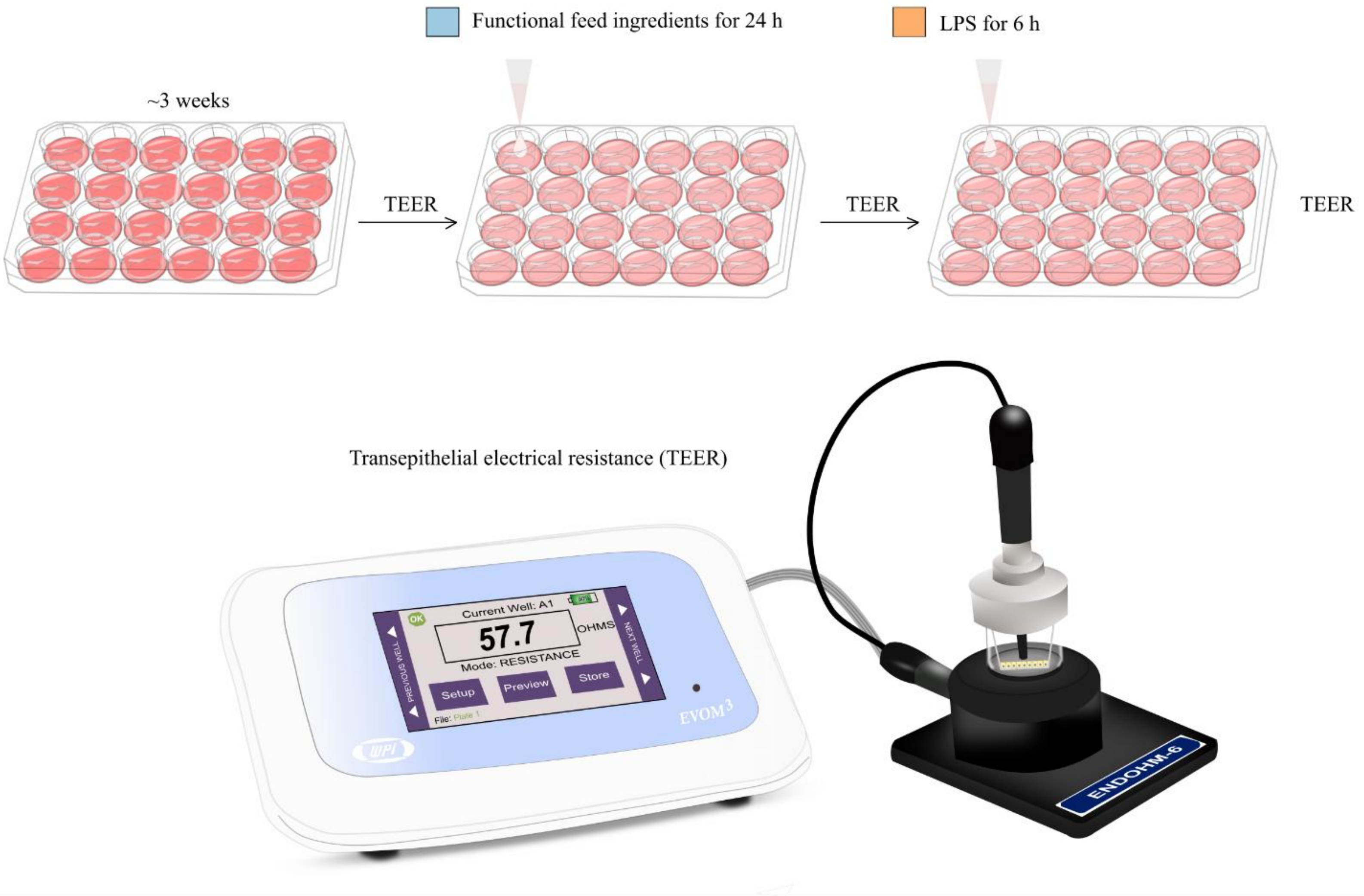

Supplementary Fig. 1. Experimental setup for measurement of transepithelial electrical resistance (TEER) in 24-well plates with 33.6 mm^2 transparent transwell inserts. TEER were performed on days 3, 7, 14 and 20 using an EVOM 3 Voltohmmeter and Endohm-6 electrode. Upon

reaching confluency, on day 20 TEER was measured, and apical media was removed and replaced with L-15/ex containing functional feed ingredients (blue color) (n=4). Following 24 hours exposure, TEER was measured again, and the cells were exposed to LPS for 6 hours (orange color) before the last TEER measurement

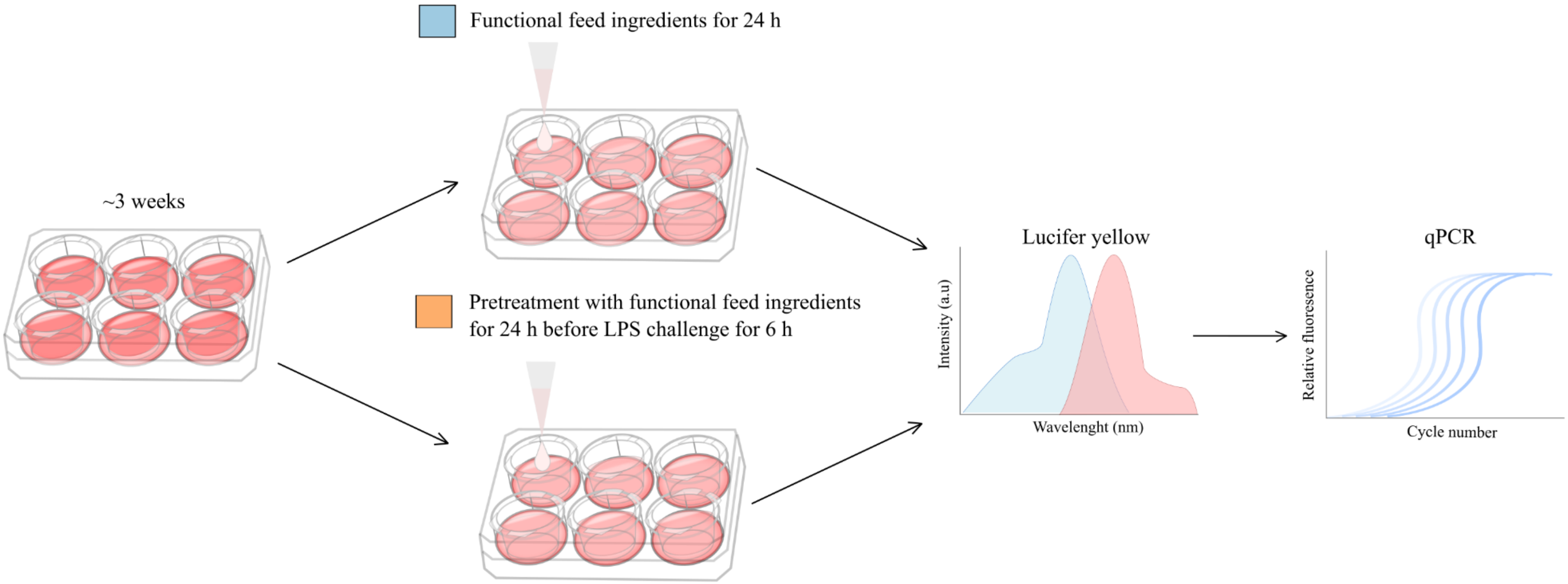

Supplementary Fig. 2. Experimental setup for Lucifer yellow (LY) translocation assay and sampling for qPCR in 6-well plates with 425.4 mm^2 transparent transwell inserts. Upon reaching confluency, on day 20, apical media was removed and replaced with L/15 ex containing functional feed ingredients (blue color) (n=6). Following 24 hours exposure, the LY translocation assay was conducted before the LY dye mix was

removed and cells washed twice with 1 mL PBS. After, the cells were sampled for qPCR analysis using TRI reagent solution (n=3). The rest of the samples that were pretreated with 24 hours exposure of functional feed ingredients was exposed to 6 hours LPS before the LY translocation assay was followed, and the cells were sampled for qPCR analysis (n=3).
