# Supplementary material for: Functional feed ingredients modulate the immune response of RTgutGC cells to LPS-induced inflammation
Source: Front Immunol. 2025 Jun 18;16:1616076. doi: 10.3389/fimmu.2025.1616076 (PMC12219272; doi:10.3389/fimmu.2025.1616076)
Supplement: Supplementary file 3 [file Table2.docx]

| **Stimulants** | **LPS^a^ concentration**  **(µg/mL)** | | **Beta-glucan^a^ concentration**  **(µg/mL)** | | **Laminarin^b^ concentration**  **(µg/mL)** | | | **Carnosine^a^ concentration**  **(mM)** | | **Salmon^a^ hydrolysate concentration (µg/mL)** | |
| --- | --- | --- | --- | --- | --- | --- | --- | --- | --- | --- | --- |
| **Working solutions** | 1 | 10 | 1 | 10 | 1 | 1 | 1 | 1 | 10 | 31.25 | 100 |
|  | 2.5 | 20 | 10 | 40 | 10 | 10 | 10 | 2.5 | 30 | 62.5 | 300 |
|  | 10 | 40 | 40 | 80 | 15 | 15 | 40 | 10 | 60 | 125 | 600 |
|  | 25 | 60 | 80 | 150 | 20 | 20 | 60 | 20 | 100 | 250 | 750 |
|  | 50 | 80 | 120 | 200 | 40 | 60 | 80 | 40 | 120 | 500 | 1000 |
|  | 100 | 120 | 250 | 250 | 60 | 100 | 120 | 60 | 150 | 1000 | 1500 |

**Supplementary Table 2. Stimulant working concentrations of functional feed ingredients and LPS**

^a^ Two independent experiments with overlapping exposure concentrations were performed

^b^ Three independent experiments with overlapping exposure concentrations were performed
