# Supplementary material for: Functional feed ingredients modulate the immune response of RTgutGC cells to LPS-induced inflammation
Source: Front Immunol. 2025 Jun 18;16:1616076. doi: 10.3389/fimmu.2025.1616076 (PMC12219272; doi:10.3389/fimmu.2025.1616076)
Supplement: Supplementary file 5 [file Table4.pdf]

Supplementary Table 4. Results from two-way ANOVA showing effect of treatment (Ctr, BG40, BG60, Lam60, Lam90) and challenge (before/after LPS challenge) for TEER, $P_{app}$ and gene expression. The table presents degrees of freedom (df), mean square (Mean sq), F-value and p-value for each factor and the interaction. Dunnett multiple comparisons test were performed within each challenge group.

| **Response factor** | **Effect** | **Df** | **Sum sq** | **Mean sq** | **F value** | ***p*** |
|---|---|---|---|---|---|---|
| TEER ($\Omega$ cm$^2$) | Treatment | 4 | 67.71 | 16.93 | 6.607 | *** |
| | Challenge | 1 | 214.60 | 214.60 | 83.756 | *** |
| | Treatment : Challenge | 4 | 46.22 | 11.56 | 4.510 | ** |
| $P_{app}$ (x $10^{-6}$ cm $s^{-1}$) | Treatment | 4 | 198.00 | 49.50 | 3.761 | ** |
| | Challenge | 1 | 3.23 | 3.23 | 0.246 | |
| | Treatment : Challenge | 4 | 46.99 | 11.75 | 0.893 | |
| il6 | Treatment | 4 | 28708 | 7717 | 20.50 | *** |
| | Challenge | 1 | 207539 | 207539 | 592.74 | *** |
| | Treatment : Challenge | 4 | 30016 | 7504 | 21.43 | *** |
| il8 | Treatment | 4 | 28.2 | 7.0 | 4.160 | * |
| | Challenge | 1 | 887.3 | 887.3 | 523.732 | *** |
| | Treatment : Challenge | 4 | 12.9 | 3.2 | 1.901 | |
| il1b | Treatment | 4 | 11308 | 327 | 3.939 | * |
| | Challenge | 1 | 66558 | 66558 | 802.113 | *** |
| | Treatment : Challenge | 4 | 1504 | 376 | 4.532 | * |
| TNFa | Treatment | 4 | 100 | 25 | 1.089 | |
| | Challenge | 1 | 12811 | 12811 | 557.222 | *** |
| | Treatment : Challenge | 4 | 116 | 29 | 1.265 | |
| myd88 | Treatment | 4 | 0.4871 | 0.1218 | 4.684 | ** |
| | Challenge | 1 | 0.6239 | 0.6239 | 23.997 | *** |
| | Treatment : Challenge | 4 | 0.4296 | 0.1074 | 4.131 | * |
| tgfb | Treatment | 4 | 0.4534 | 0.11336 | 5.388 | ** |
| | Challenge | 1 | 0.0788 | 0.07877 | 3.744 | . |
| | Treatment : Challenge | 4 | 0.0352 | 0.00880 | 0.418 | |
| pcna | Treatment | 4 | 0.1016 | 0.0252 | 3.143 | * |
| | Challenge | 1 | 1.5960 | 1.5960 | 197.544 | *** |
| | Treatment : Challenge | 4 | 0.0048 | 0.0012 | 0.148 | |
| ialp | Treatment | 4 | 1.0916 | 0.27290 | 29.145 | *** |
| | Challenge | 1 | 0.0057 | 0.00574 | 0.613 | |
| | Treatment : Challenge | 4 | 0.1260 | 0.03149 | 3.363 | * |
| cdh1 | Treatment | 4 | 0.3701 | 0.0925 | 6.493 | ** |
| | Challenge | 1 | 3.1440 | 3.1440 | 220.611 | *** |
| | Treatment : Challenge | 4 | 0.1072 | 0.0268 | 1.880 | |
| cldn3 | Treatment | 4 | 0.1428 | 0.0357 | 1.596 | |
| | Challenge | 1 | 0.9976 | 0.9976 | 44.591 | *** |
| | Treatment : Challenge | 4 | 0.2695 | 0.0674 | 3.011 | * |
| cldn12 | Treatment | 4 | 0.7292 | 0.18231 | 20.345 | *** |
| | Challenge | 1 | 0.0489 | 0.04893 | 5.460 | * |
| | Treatment : Challenge | 4 | 0.0680 | 0.01701 | 1.898 | |
| zo-1 | Treatment | 4 | 0.2194 | 0.0548 | 3.731 | * |
| | Challenge | 1 | 0.4021 | 0.4021 | 27.356 | *** |
| | Treatment : Challenge | 4 | 0.1979 | 0.0495 | 3.366 | * |
| | Treatment | 4 | 0.7801 | 0.19503 | 9.580 | *** |

| | | | | | | |
|---|---|---|---|---|---|---|
| slc10a2 | Challenge<br>Treatment : Challenge | 1<br>4 | 0.0655<br>0.0571 | 0.06550<br>0.01429 | 3.218<br>0.702 | . |
