# Supplementary material for: Functional feed ingredients modulate the immune response of RTgutGC cells to LPS-induced inflammation
Source: Front Immunol. 2025 Jun 18;16:1616076. doi: 10.3389/fimmu.2025.1616076 (PMC12219272; doi:10.3389/fimmu.2025.1616076)
Supplement: Supplementary file 6 [file Table5.pdf]

Supplementary Table 5. Results from two-way ANOVA showing effect of treatment (Ctr, Carn100, Carn120, FPH300, FPH600) and challenge (before/after LPS challenge) for TEER, P_{app} and gene expression. The table presents degrees of freedom (df), mean square (Mean sq), F-value and p-value for each factor and the interaction. Dunnett multiple comparisons test were performed within each challenge group.

**Response factor**	**Effect**	**Df**	**Sum sq**	**Mean sq**	**F value**	***p***
TEER (Ω cm^2)	Treatment	4	71.6	17.9	5.978	**
	Challenge	1	318.3	318.3	106.312	***
	Treatment : Challenge	4	90.9	22.7	7.587	***
P_{app} (x 10^{-6} cm s^{-1})	Treatment	4	86.3	21.59	1.339	
	Challenge	1	193.9	193.95	12.031	**
	Treatment : Challenge	4	120.4	30.11	1.868	
il6	Treatment	4	125854	31463	7.468	***
	Challenge	1	294302	294302	69.855	***
	Treatment : Challenge	4	133516	33379	7.923	***
il8	Treatment	4	404.0	101.0	7.543	***
	Challenge	1	1463.9	1463.9	109.326	***
	Treatment : Challenge	4	624.7	156.2	11.664	***
il1b	Treatment	4	51456	12864	6.697	**
	Challenge	1	143674	143674	74.793	***
	Treatment : Challenge	4	38419	13740	7.153	***
TNFa	Treatment	4	4130	1032	6.644	**
	Challenge	1	15294	15294	98.424	***
	Treatment : Challenge	4	4893	4893	7.873	***
myd88	Treatment	4	0.9121	0.2280	8.247	***
	Challenge	1	0.7603	0.7603	27.501	***
	Treatment : Challenge	4	1.5934	0.3983	14.408	***
tgfb	Treatment	4	2.9455	0.7364	4.680	**
	Challenge	1	0.0014	0.0014	0.009	
	Treatment : Challenge	4	0.9050	0.2263	1.438	
pcna	Treatment	4	1.9932	0.4983	48.67	***
	Challenge	1	0.5643	0.5643	55.12	***
	Treatment : Challenge	4	0.7210	0.1802	17.61	***
ialp	Treatment	4	0.4816	0.12041	5.121	**
	Challenge	1	0.2160	0.21601	9.186	**
	Treatment : Challenge	4	0.2018	0.05046	2.146	
cdh1	Treatment	4	3.015	0.7539	13.991	***
	Challenge	1	2.293	2.2926	42.549	***
	Treatment : Challenge	4	0.982	0.2455	4.556	**
cldn3	Treatment	4	1.6286	0.4071	27.20	***
	Challenge	1	1.3107	1.3107	87.58	***
	Treatment : Challenge	4	1.1955	0.2989	19.97	***
cldn12	Treatment	4	0.2258	0.05644	1.820	
	Challenge	1	0.1332	0.13316	4.295	.
	Treatment : Challenge	4	0.7665	0.19163	6.181	**
zo-1	Treatment	4	0.2640	0.06600	4.782	**
	Challenge	1	0.2465	0.24650	17.860	***
	Treatment : Challenge	4	0.2236	0.05591	4.051	*
	Treatment	4	1.9281	0.4820	7.065	**

slc10a2	Challenge<br>Treatment : Challenge	1<br>4	0.0031<br>0.1384	0.0031<br>0.0346	0.045<br>0.507	
